# Activation of human mast cells by retrocyclin and protegrin highlight their immunomodulatory and antimicrobial properties

**DOI:** 10.18632/oncotarget.5611

**Published:** 2015-09-10

**Authors:** Kshitij Gupta, Akhil Kotian, Hariharan Subramanian, Henry Daniell, Hydar Ali

**Affiliations:** ^1^ Department of Pathology, School of Dental Medicine, University of Pennsylvania, Philadelphia, PA, USA; ^2^ Department of Biochemistry, School of Dental Medicine, University of Pennsylvania, Philadelphia, PA, USA

**Keywords:** antimicrobial peptides, mast cells, retrocyclin, protegrin, chloroplast, MrgX2, Immunology and Microbiology Section, Immune response, Immunity

## Abstract

Preclinical evaluation of Retrocyclins (RC-100, RC-101) and Protegrin-1 (PG-1) antimicrobial peptides (AMPs) is important because of their therapeutic potential against bacterial, fungal and viral infections. Human mast cells (HMCs) play important roles in host defense and wound healing but the abilities of retrocyclins and protegrin-1 to harness these functions have not been investigated. Here, we report that chemically synthesized RC-100 and PG-1 caused calcium mobilization and degranulation in HMCs but these responses were not blocked by an inhibitor of formyl peptide receptor-like 1 (FPRL1), a known receptor for AMPs. However, RC-100 and PG-1 induced degranulation in rat basophilic leukemia (RBL-2H3) cells stably expressing Mas related G protein coupled receptor X2 (MrgX2). Chemical synthesis of these AMPs is prohibitively expensive and post-synthesis modifications (cyclization, disulfide bonds, folding) are inadequate for optimal antimicrobial activity. Indeed, we found that synthetic RC-100, which caused mast cell degranulation via MrgX2, did not display any antimicrobial activity. Green-fluorescent protein (GFP)-tagged RC-101 (analog of RC-100) and GFP-tagged PG-1 purified from transgenic plant chloroplasts killed bacteria and induced mast cell degranulation. Furthermore, GFP-PG1 bound specifically to RBL-2H3 cells expressing MrgX2. These findings suggest that retrocyclins and protegrins activate HMCs independently of FPRL1 but via MrgX2. Harnessing this novel feature of AMPs to activate mast cell's host defense/wound healing properties in addition to their antimicrobial activities expands their clinical potential. Low cost production of AMPs in plants should facilitate their advancement to the clinic overcoming major hurdles in current production systems.

## INTRODUCTION

Antibiotics have been used for treatment of microbial infections since the early 1900s but emergence of multidrug resistant strains of microbes poses a tremendous public health concern globally [1]. Thus, there is an urgent need to develop novel therapy for the treatment of infectious diseases caused by antibiotic resistant organisms. Cationic antimicrobial peptides (AMP), also known as host defense peptides (HDPs) such as the cathelicidin LL-37 and human β-defensins (hBDs) have the potential to be utilized as antimicrobial agents [2–4]. In addition to their direct antimicrobial activity, LL-37 and hBDs display immunomodulatory properties which include the recruitment and activation of immune cells such as mast cells, neutrophils, monocytes and T cells [5–9]. These AMPs also modulate angiogenesis and promote wound healing [10–12]. In addition, many AMPs cause red blood cell lysis and display cytotoxic activity against immune and non-immune cells [13, 14]. Great strides have been made in recent years in identifying and optimizing AMPs for lower toxicity with greater stability/activity, which can also harness the immune system for therapeutic benefits [15, 16].

A family of AMPs known as θ-defensins is found in leukocytes of rhesus macaques [17]. These cyclic octadecapeptides are stabilized by three disulfide bonds, display antifungal, antibacterial, antiviral activities and are thought to play an important role in host defense in primates [18]. Although mRNA transcripts for θ-defensins are found in humans, corresponding peptides are not expressed due to a premature stop codon which prevents their translation [19]. It has been proposed that this mutation and the resulting absence of θ-defensins may render humans more susceptible to human immunodeficiency virus (HIV) infection than rhesus macaques [20]. Retrocyclin-1 (RC-100) is a cyclic octadecapeptide humanized θ-defensin, which was initially prepared by solid phase synthesis [19]. It has broad spectrum antimicrobial properties and protects human target cells from HIV-infection *in vitro* [19]. This protective effect does not involve direct inactivation of the virus but reflects high affinity binding to gp120 and galactosylceramide [21]. An analog of RC-100 containing a single arginine to lysine substitution (RC-101) has greater antimicrobial and anti-HIV effects [22]. Unlike hBD3 and LL-37, retrocyclins are non-hemolytic and non-cytotoxic but whether they activate immune cells has not yet been determined [23].

Protegrin-1 (PG-1) is an antimicrobial peptide that was originally isolated from porcine leukocytes [24]. It shares many structural similarities with θ-defensin; it is a cysteine rich octadecapeptide with high arginine content but lacks a cyclic backbone [18]. The anti-parallel β-hairpin conformation of PG-1 is stabilized by two cysteine-cysteine disulfide bonds and contributes substantially to their antimicrobial activity [24–27]. Due to the unique structure and broad-spectrum antimicrobial activities, retrocyclins and PG-1 have immense therapeutic potential against infectious diseases. A major limitation of chemically synthesized peptides is that they are prohibitively expensive (∼$600,000 - $700,000/gram). In addition, post-synthesis modifications (cyclization, disulfide bonds and folding) are less than adequate for their optimal antimicrobial activity. Most commercial sources of retrocyclin have no antimicrobial activity due to inadequate cyclization. To overcome these limitations, we have expressed RC-101 and PG-1 in transgenic tobacco chloroplasts as GFP-fusion proteins. Both these AMPs are folded properly with suitable posttranslational modifications (cyclization and disulfide bonds) and have potent antimicrobial activity against bacterial and viral pathogens [28].

Currently around 500 – 600 AMP drugs are in clinical trials as a result of their high efficacy, pathogenic specificity and safety shown in *in vitro* experiments [29]. After establishing the *in vitro* efficacy of RC-101 against various pathogens, formulated peptide has been shown to be efficacious in several primate and human *ex vivo* tissue culture models [22, 30]. RC-101 is also effective *in vivo* when applied as a topical microbicide on vaginal tissue in a pigtailed macaque model [31]. The preclinical safety shown by this AMP has made it a promising candidate to move ahead with safety trials in humans. In the case of PG-1, Iseganan a synthetic analogue of protegrin has been developed as an oral mouthwash against opportunistic pathogens and has already been tested in several Phase II and Phase III clinical trials [32–34]. Before further trials are carried out, it is important to mechanistically understand the impact of AMPs on non-target cells, especially immunomodulatory cells in addition to their effect on microbes.

Mast cells are multifunctional immune cells found in all mammalian vascularized tissues, most commonly at sites exposed to the external environment, such as the skin, oral mucosa, airway and intestine. Not surprisingly, mast cells play a sentinel role in host defense, orchestrate innate immunity and promote wound healing [35–44]. Mas-related G protein coupled receptor-X2 (MrgX2) was originally identified as a novel G protein coupled receptor (GPCR) that is expressed in the dorsal root ganglia and participates in the perception of pain [45]. Outside the dorsal root ganglia, the expression of this receptor is restricted to human mast cells and no other immune or structural cells [46, 47]. We have recently shown that the AMPs such as human β-defensins and the cathelicidin LL-37 activate human mast cells via MrgX2 to induce G protein-mediated Ca^2+^ mobilization and robust mast cell degranulation [6, 7]. Unlike MrgX2, FPRL1 (also known as FPR2), a member of the chemokine GPCRs, is expressed in a variety of cells including mast cells, neutrophils, macrophages and ovarian cancer cells [8, 48, 49]. Mast cells are the only immune cells that are known to express both MrgX2 and FPRL1. Furthermore, AMPs such as hBD3 and LL-37 activate human mast cells via MrgX2 but pleurocidin does so via FPRL1 [6, 7, 50]. These findings raise the interesting possibility that RC-100/RC-101 and PG-1 could activate human mast cells via MrgX2 or FPRL1, thereby contributing to their therapeutic potentials as antimicrobial agents.

With the exception of our recent reports on human defensins, none of the AMPs in clinical development have been investigated for their role in immune modulation via mast cell activation. Here, we report that two AMPs (retrocyclin and protegrin) currently in clinical development activate human mast cells via a mechanism different from human HDPs (independent of FPRL1) but through the same receptor (MrgX2). Our studies also demonstrate a dissociation of synthetic RC-100's ability to activate mast cells from its antimicrobial activity and could reflect the peptide's cyclization status. Unlike most GPCRs that are expressed in mast cells, MrgX2 is located at both plasma membrane and intracellular sites [6, 47] but the relative contribution of these receptors on mast cell activation is unknown. The availability of chloroplast expressed GFP-tagged PG-1 allowed us to demonstrate that activation of cell surface MrgX2 by this AMP is sufficient to cause mast cell degranulation. Increased understanding of AMPs’ mechanism of action, their interaction with non-target cells and low cost production should facilitate further clinical development.

## RESULTS

### PG-1 and RC-100 induce degranulation in HMCs but RC-100 does not have antimicrobial activity

**Figure 1:**
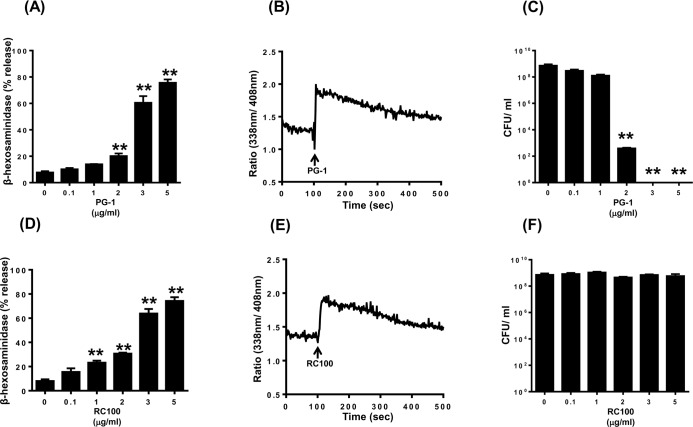
PG-1 and RC-100 induce degranulation and Ca^2+^ mobilization in LAD2 human mast cells; however have differential antimicrobial activities LAD2 mast cells were stimulated with different concentrations of **A.** PG-1 or **D.** RC-100 and percent degranulation (β-hexosaminidase release) was determined. Data are mean ± SEM of three experiments. LAD2 cells were loaded with Indo-1AM and Ca^2+^ mobilization in response **B.** PG-1 or **E.** RC-100 (both 3 μg/ml) was determined. To test the biological activity of the commercial peptides a starting inoculum of 4 × 10^5^ CFU/ml of *E. coli* was incubated in the presence of **C.** PG-1 and **F.** RC-100 at the indicated concentrations for a period of 8 hours, after which the cultures were plated on LB Agar and incubated overnight. The colony forming units were counted the next day to identify the survival rate of *E. coli*. Data shown are representative of 3 similar experiments. Statistical significance was determined by two-way ANOVA with Bonferroni's post test. ** indicates *p* < 0.001.

We used laboratory of allergic diseases 2 (LAD2) cells to determine the effects of PG-1 and RC-100 on mast cell signaling and degranulation. We found that PG-1 (2 μg/ml) induced significant degranulation and a maximal response of ∼80% was observed at a concentration of 5 μg/ml (Figure 1A). Because increase in intracellular Ca^2+^ mobilization provides an important signal for mast cell degranulation, we sought to determine the effect of PG-1 on this response. We found that PG-1 (3 μg/ml), which caused ∼60% mast cell degranulation, was associated with a sustained Ca^2+^ response (Figure 1B). To determine the relationship between mast cell activation and antimicrobial activity, we tested the effects of different concentrations of PG-1 on bacterial growth. As shown in Figure 1A and 1C, PG-1 caused mast cell degranulation and inhibited bacterial growth at a similar concentration range (2–5 μg/ml).

RC-100 also induced degranulation and Ca^2+^ mobilization in human mast cells and it was more potent than PG-1 (Figure 1D and 1E). Thus, while PG-1 (1 μg/ml) did not induce mast cells degranulation, RC-100 at this concentration induced a significant response. Despite this difference, both peptides induced a similar maximal response (∼80% degranulation) at a concentration of 5 μg/ml. Surprisingly, RC-100 did not prevent bacterial growth even at a high concentration (5 μg/ml) (Figure 1F), which induced substantial mast cell degranulation (Figure 1D).

### Roles of G proteins on PG-1 and RC-100-induced Ca^2+^ mobilization and degranulation in human mast cells

**Figure 2:**
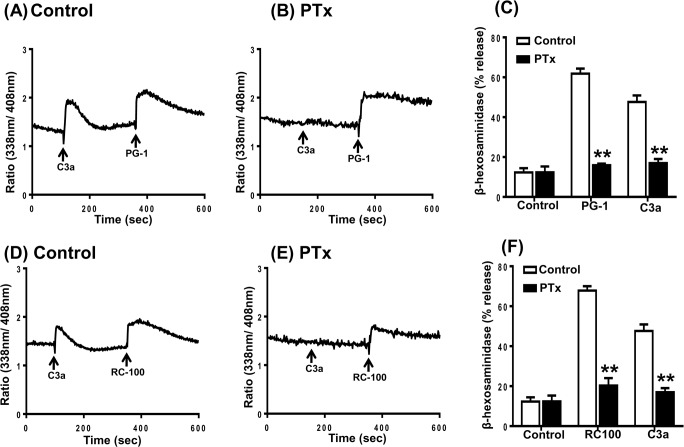
Effects of Pertussis toxin on C3a, PG-1 and RC-100-induced Ca^2+^ mobilization and degranulation in human mast cells **A.**, **D.** Indo-1 loaded LAD2 cells were exposed to C3a (1 nM), followed by PG-1 (3 μg/ml) or RC-100 (3 μg/ml) and intracellular Ca^2+^ mobilization was determined. **B.**, **E.** Cells were treated with pertussis toxin (PTx; 100 ng/ml, 16 h) and effects of C3a, PG-1 or RC-100 on Ca^2+^ mobilization was determined. **C.**, **F.** Cells were exposed to Pertussis toxin (PTx; 100 ng/ml, 16h) and C3a, PG-1 and RC-100-induced degranulation was determined. Data are mean ± SEM of three experiments. Statistical significance was determined by two-way ANOVA with Bonferroni's post test. and ** indicates *p* < 0.001.

Next, we sought to determine the involvement of G proteins on mast cell responses to PG-1 and RC-100. Pertussis toxin (PTx), an inhibitor of Gαi-family of G protein, is known to block the complement component C3a-induced Ca^2+^ mobilization and degranulation in human mast cells [51, 52]. While PTx completely blocked C3a-induced Ca^2+^ mobilization it had little or no effect on the responses elicited by either PG-1 or RC-100 (Figure 2A, 2B, 2D and 2E). Notably, treatment of cells with PTx almost completely blocked degranulation induced by PG-1 and RC-100 (Figure 2C and 2F). These findings demonstrate that PG-1 and RC-100 cause degranulation in human mast cells via the interaction of a Gαi-independent Ca^2+^ influx and an unknown Gαi-dependent signaling pathway.

### PG-1 and RC-100 mediated degranulation in HMCs does not involve FPRL1

**Figure 3:**
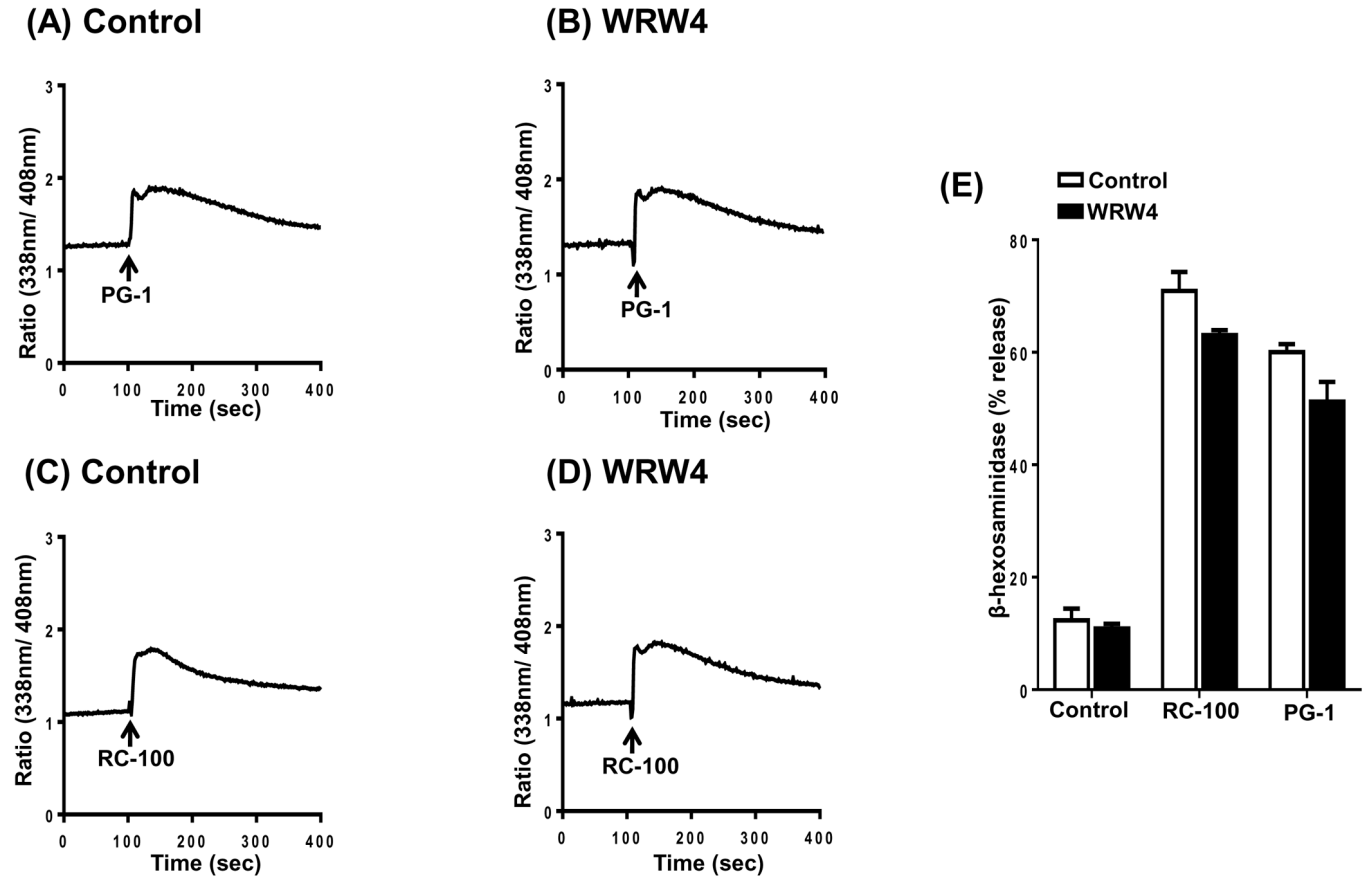
PG-1 and RC-100 induced Ca^2+^ mobilization and degranulation are independent of FPRL1 Indo-1 loaded LAD2 cells were preincubated with buffer (Control) or WRW4 (10 μM) for 30 min and Ca^2+^ mobilization was determined in response to **A.**, **B.** PG-1 (3 μg/ml) or **C.**, **D.** RC-100 (3 μg/ml). **E.** LAD2 cells were pretreated with buffer (Control) or WRW4 (10 μM) and stimulated with 3 μg/ml concentration of PG-1 and RC-100 and percent degranulation was determined. Data are represented as mean ± SEM of three experiments.

The cathelicidin antimicrobial peptide LL-37 activates human neutrophils and monocytes via the utilization of FPRL1 [8]. Human mast cells also express FPRL1 and the cationic AMP pleurocidin induces mast cell via this receptor [50]. Furthermore, a selective FPRL1 antagonist peptide, WRW4 blocks pleurocidin-induced mast cell degranulation [50]. To determine the possible role of FPRL1 on PG-1 and RC-100-induced responses in HMCs, we utilized WRW4. We found that pretreatment of cells with WRW4 had no effect on PG-1 and RC-100 induced Ca^2+^ mobilization (Figure 3A–3D) or degranulation (Figure 3E). These findings suggest that FPRL1 does not participate in RC-100 or PG-induced mast cell responses.

### PG-1 and RC-100 activate human mast cells via MrgX2

**Figure 4:**
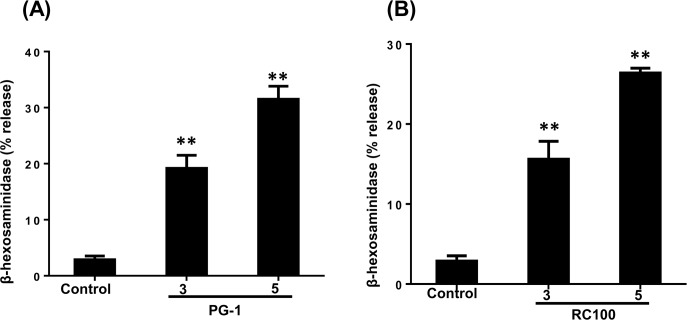
PG-1 and RC-100 induce degranulation in RBL-2H3 cells expressing MrgX2 **A.**, **B.** RBL-2H3 cells stably expressing MrgX2 were exposed to buffer (Control), PG-1 or RC-100 (3 μg/ml, 5 μg/ml) for 30 min and β-hexosaminidase release was measured. Data are represented as mean ± SEM of three experiments. Statistical significance was determined by one-way ANOVA with Bonferroni's post test. * indicates *p* < 0.01 and ** indicates *p* < 0.001.

Considering the recent demonstration that MrgX2 acts as the receptor for a range of cationic peptides including AMPs [6, 7, 46, 53], we hypothesized that PG-1 and RC-100 could activate HMCs via this receptor. RBL-2H3 cell is a rat basophilic leukemia cell line that has been extensively used to study the role of IgE receptor (FcεRI) and GPCR signaling in mast cells [51, 54, 55]. We have recently shown that RBL-2H3 cells are unresponsive to human AMPs unless the cells are transfected with cDNA encoding MrgX2 [56]. To determine the role of MrgX2 on PG-1 and RC-100-induced degranulation, we utilized RBL-2H3 cells stably expressing human MrgX2. In this system, PG-1 and RC-100 induced substantial mast cell degranulation (Figure 4A and 4B). Taken together, these data demonstrate that PG-1, RC-100 activate mast cells via MrgX2.

### Expression and purification of GFP-tagged RC-101 and PG-1 from transgenic plants

**Figure 5:**
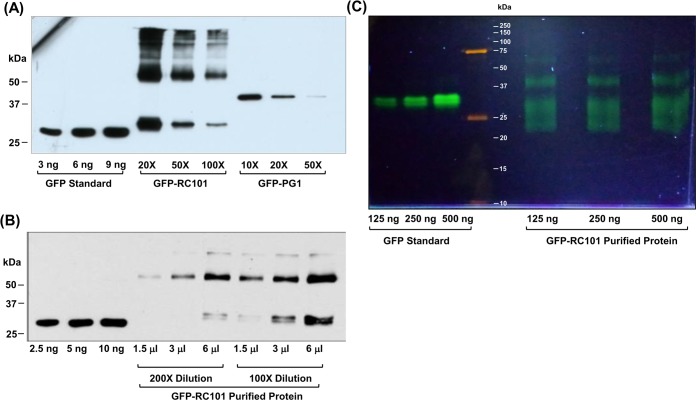
Quantification and evaluation of GFP-RC101 and GFP-PG1 expressed in transgenic plants **A.** Western blot analysis of GFP-RC101 and GFP-PG1 protein extracts from the transgenic tobacco leaves. Total Soluble Protein was loaded at indicated dilutions. **B.** Western blot quantification of GFP- RC101 purified from transgenic tobacco leaves. Purified protein was loaded at the indicated volumes of 100X and 200X dilutions. **C.** Fluorescence of GFP-RC101 protein is maintained after column purification as seen in non denaturing native PAGE (12%), observed under UV light.

To determine the expression levels of GFP-RC101 and GFP-PG1 in transgenic plants, plant extracts were quantified by western blotting using anti-GFP antibody (Figure 5A). Expression level of GFP-RC101 was between 20 – 34% and GFP-PG1 was 4 – 8% of total leaf protein. One gram of fresh leaf yielded about 100 μg of GFP protein on an average with an overall recovery of 20% and 95% purity. Figure 5B shows the purified GFP-RC101 obtained after dialysis, lyophilization and reconstitution in a small volume of phosphate-buffered saline (PBS) and quantified by immunoblotting against GFP. GFP-RC101 (29 kDa) purified protein has a slightly higher molecular weight than GFP standard (27 kDa) due to RC101 fusion (1.9 kDa). The purity of such preparations was about 90% when evaluated through immunoblot analysis. To evaluate purity and functionality of GFP-RC101, the purified proteins were run under non-denaturing conditions in a native PAGE. Figure 5C shows purified GFP-RC101 exhibiting increasing fluorescence intensity corresponding to the amount of total protein loaded, confirming GFP functionality. The distinct bands observed under non-denaturing conditions are due multimerization of the GFP-RC101 and differential electrical mobility due to the high positive charge present on RC101 and secondary structures.

### GFP-RC101 and GFP-PG1 isolated from plant chloroplast display antimicrobial activity and cause mast cell degranulation via MrgX2

**Figure 6:**
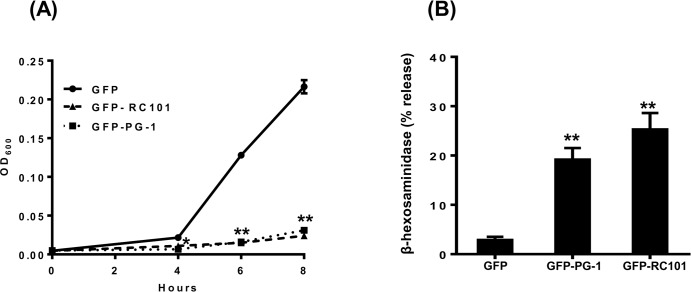
Antimicrobial and immunomodulatory effects of AMPs produced in plants **A.** Antimicrobial activity assay of AMPs purified from transplastomic plants against *E. coli*. The inhibitory effect of the AMPs on the growth of *E. coli* (starting inoculum 4 × 10^5^ CFU/ml) was measured by monitoring OD_600_ for 8 h in the presence of vehicle control (GFP), GFP-RC101 (0.8 μg/ml) or GFP-PG1 (0.5 μg/ml). **B.** LAD2 cells were exposed to vehicle control (GFP), GFP-RC101 (1.8 μg/ml) or GFP-PG1 (2.4 μg/ml) and percent degranulation (β-hexosaminidase release) was determined 30 min after stimulation. Data are represented as mean ± SEM of three experiments. Statistical significance was determined by one-way ANOVA with Bonferroni's post test. * indicates *p* < 0.01 and ** indicates *p* < 0.001.

To determine antimicrobial activity of chloroplast-derived GFP-RC101 and GFP-PG1, we tested their effects on growth of *E. coli*. As shown in Figure 6A, both peptides completely inhibited growth of *E. coli*. To determine their effects on mast cell degranulation, we incubated LAD2 mast cells with GFP-RC101 (1.8 μg/ml) and GFP-PG1 (2.4 μg/ml). We found that GFP-PG1 and GFP-RC101 cause significant degranulation of human mast cells (Figure 6B) and such responses were equivalent to those elicited by chemically synthesized commercial peptides (Figure 1A and 1D).

**Figure 7:**
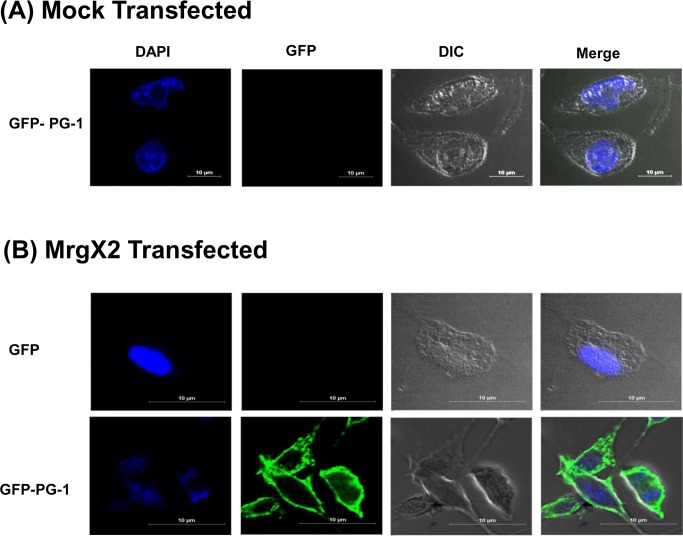
GFP-PG1 associate with the plasma membrane of MrgX2-expressing RBL-2H3 cells **A.** Mock transfected RBL-2H3 cells were exposed to GFP-PG1 (4 μg/ml, 30 min) and **B.** RBL-2H3 cells transfected with MrgX2 were exposed to either GFP or GFP-PG1 (4 μg/ml, 30 min). Cells were washed with ice cold PBS, fixed in 4% paraformaldehyde, nuclei were counter-stained with 4′-6-diamidino-2-phenylindole dihydrochloride (DAPI) and observed using laser scanning confocal microscope (Nikon A1R) with a 60x water objective and 5.6X digital zoom. Images were analyzed using Nikons Elements 4.1 software and representative images from 25 stored images for each condition are shown.

An important property of MrgX2 that distinguishes it from other GPCRs in mast cells is that this receptor is located at both plasma membrane and intracellular sites [6, 47]. This raises an interesting possibility that PG-1 or RC-101 could activate mast cells via their interaction with cell surface or intracellular receptors. To test this possibility, we exposed mock or MrgX2-transfected RBL-2H3 cells to GFP alone or GFP-PG1 for 30 min and observed GFP fluorescence in mast cells by confocal microscopy. As shown in Figure 7A, GFP-PG1 did not associate with mock-transfected RBL-2H3 cells. In contrast, GFP-PG1 was associated with the plasma membrane of MrgX2-expressing cells but not GFP alone without PG1 fusion (Figure 7B). These findings suggest that GFP-PG1 induces mast cell degranulation via the activation of MrgX2 receptors that are present on the cell surface.

## DISCUSSION

Retrocyclins and protegrins are cyclic or acyclic peptides with potent antimicrobial activities against a broad spectrum of microorganisms, including bacteria, fungi, yeast. They also bind to glycoprotein 120 to prevent HIV entry. These peptides therefore have potential for use as anti-microbial and anti-HIV agents. Many AMPs not only display antimicrobial activity but they also have immunomodulatory and wound healing properties that can be harnessed for their therapeutic activity. However, to the best of our knowledge, the effects of retrocyclins and protegrins on the activation of immune cells such as mast cells have never been tested.

An important limitation of studies with these peptides is the tremendously high cost of their chemical synthesis ($600,000 - $700,000/gram). In addition, post-synthesis modifications (cyclization, disulfide bonds and folding) are less than adequate for optimal antimicrobial activity. Thus, most commercial source of retrocyclin has no or negligible antimicrobial activity due to inadequate cyclization or other post-translational modifications. Synthesis of recombinant AMPs in microbial systems is challenging because of their antimicrobial activity. Therefore, in this study, we compared both chemically synthesized and recombinant GFP-tagged peptides expressed in chloroplasts and evaluated their antimicrobial and immunomodulatory properties. Chloroplasts are prokaryotic compartments within eukaryotic cells with exceptional ability to synthesize foreign proteins from different kingdoms including bacterial [57] viral [58] fungal [59], animal [60] and human genes [61–65]. Because of expression of >10,000 copies of transgenes in each plant cell, foreign proteins are expressed at very high levels (up to 70% of total leaf protein). Expressed recombinant proteins can be indefinitely stored at ambient temperature in lyophilized cells maintaining their efficacy and functionality [66, 67], thereby eliminating expensive cold storage/transportation and short shelf life. These are highly desired features for affordable production and delivery of human therapeutic proteins in the clinic.

Most importantly, chloroplasts perform all post-translational modifications required for fully functional antimicrobial peptides including folding, stabilization with disulfide bond and cyclization. Several studies have shown that human blood proteins (like insulin, interferon, etc), when expressed in chloroplasts are properly folded with disulfide bonds and are fully functional upon oral or injectable delivery [68, 69]. Chloroplasts also assemble complex multimeric structures (like cholera toxin B) with disulfide bonds that bind to epithelial cell receptors like GM1 (General Monosialotetrahexosylganglioside) [60, 61, 64, 70–73]. Assembly of virus-like particles that are required for stability and efficacy of vaccine antigens has been observed in chloroplasts [74, 75]. Protein disulfide isomerase/thioredoxin expression has been shown to enhance folding and assembly of human serum albumin within chloroplasts, a complex protein requiring 17 disulfide bonds [76]. Cyclization with disulfide bonds is required for antimicrobial activity of retrocyclin and chloroplasts synthesize and fold such cyclic proteins [28]. Functional evaluation of both protegrin and retrocyclin synthesized in chloroplasts, in this study, again confirms proper folding and formation of disulfide bonds.

Human mast cells express FPRL1 and MrgX2 and these GPCRs are activated by different AMPs [6, 7, 50]. We found that a potent and selective inhibitor of FPRL1 had no effect on RC-100 or PG-1-induced Ca^2+^ mobilization and degranulation. Furthermore, expression of MrgX2 in a rodent mast cell line RBL-2H3 cells, which do not endogenously express this receptor, rendered these cells responsive to both RC-100 and PG-1 for degranulation. These findings demonstrate that RC-100 and PG-1 bind to MrgX2 receptor to induce signaling and degranulation in human mast cells. It is noteworthy that in unstimulated mast cells, MrgX2 is located at both plasma membrane and intracellular sites [6, 47]. There is now growing body of evidence for the role of both plasma membrane and intracellular GPCRs for sustained signaling and effector function [77]. Substance P, which couples to MrgX2, has been shown to activate intracellular G proteins to induce mast cell degranulation [46, 78]. This raises the interesting possibility that AMPs used in this study could induce mast cell degranulation via the activation of intracellular MrgX2. The availability of GFP-PG1 allowed us to test this possibility. The findings that GFP-PG1 interacted with RBL-2H3 transiently expressing MrgX2 but not mock transfected cells clearly demonstrated a specific ligand-receptor interaction. We found that the interaction of GFP-PG1 with MrgX2-expressing RBL-2H3 cells was localized to the plasma membrane and no green fluorescence was detected at intracellular sites even after prolonged incubation. This finding demonstrates that GFP-PG1 interacts with cell surface MrgX2 on mast cells to induce signaling and degranulation.

One important finding of the present study is that while the synthetic RC-100 activated mast cells via MrgX2, it had no detectable antimicrobial activity. The data sheet from the manufacturer indicated that the peptide is >95% pure and has the appropriate molecular mass but did not provide evidence for antimicrobial activity. It has previously been shown that while structures and stabilities of retrocyclins depend on the number and position of the disulfide bonds, their antimicrobial and membrane binding properties depend on the presence of cyclic backbone [21]. Our previous studies have shown that both linear and cyclic cationic peptides including substance P, hBD3 and LL-37 activate human mast cells via MrgX2 [6, 7, 56]. Thus, it is likely that the cationic residues on AMPs such as hBD3, LL-37, retrocyclins and protegrins interact with negatively charged residues on MrgX2 to induce mast cell activation but their ability to cause microbial killing requires additional posttranslational modifications (cyclization, disulfide bonds, folding). These findings suggest that different structural determinants on AMPs dictate their antimicrobial and immunomodulatory properties. Thus, new molecules can be designed in the future to modulate their different functions.

Unlike circulating leukocytes, mast cells are tissue resident cells that are found beneath the epithelium and close to blood vessels. The epithelium expresses pattern recognition receptors (e.g. toll-like receptors), which responds to infectious agents to generate AMPs [79, 80]. These cationic peptides kill microbes by binding to negatively charged residues on their membrane [81]. Mast cell degranulation plays an important role in innate immunity by causing increased vascular permeability and by initiating the recruitment of neutrophil to the sites of infection [36–39, 43, 44]. We have previously shown that hBD3, which is derived from epithelial cells, activates human mast cell via MrgX2 [7]. Furthermore, hBD3 increases vascular permeability in a mast cell-dependent manner [82]. These findings suggest that MrgX2 expressed in mast cells contributes to innate immunity via its effects on vascular permeability and neutrophil recruitment.

In addition to innate immunity, mast cells orchestrate the development of adaptive immunity and play an important role in wound healing [35, 40–42]. Thus, at sites of microbial infection, mediators released from mast cells promote migration of dendritic cells, which are subsequently increased in draining lymph nodes [83–85]. Furthermore, mast cell-derived histamine directly modulates dendritic cell activation to enhance antigen presentation to T cells [86]. Interestingly, compound 48/80, which activates mast cells via MrgX2, has been used as a safe and effective vaccine adjuvant in mice [42, 46, 87]. Wound healing is a dynamic process that involves a number of overlapping phases including inflammation, granulation tissue formation, wound contraction and remodeling. Wound provides an excellent breeding ground for microbes and proper healing is dependent on managing the microbial burden. Interestingly, LL-37 and hBD3, which display antimicrobial activity and cause mast cell degranulation via MrgX2, also promote wound healing [6, 11, 12, 88]. It is noteworthy that mast cells are only immune cells that express MrgX2 and the data presented herein demonstrate that retrocyclins and PG-1 activate mast cells via this receptor. These findings suggest that potential therapeutic benefits of retrocyclin/PG-1 and other AMPs *in vivo* not only reflects their antimicrobial activity but also involves the harnessing of mast cell's immunomodulatory and wound healing properties.

In conclusion, biopharmaceuticals produced in current systems are prohibitively expensive and are not affordable for a large majority of the global population. Chemically synthesized AMPs used in this study cost $600,000 - $700,000 per gram and their post-synthesis modifications (cyclization, disulfide bonds, folding) are inadequate for optimal antimicrobial activity (retrocyclin 100 has no detectable antimicrobial activity). Therefore, in this report we synthesized fully functional AMPs in plant chloroplasts, making them affordable for the large global population who often face outbreak of infectious diseases. Enhancing our understanding of mechanistic aspects of AMPs is important for clinical development. With the exception of our recent reports on human HDPs, none of the AMPs in clinical development have been investigated for their role in immune modulation via mast cell activation. Here, we report the novel finding that two AMPs (retrocyclin and protegrin) currently in clinical development as antimicrobial agents activate human mast cells via MrgX2. Using AMPs with GFP fusion expressed in plant chloroplasts we demonstrate that activation of cell surface receptor is sufficient to cause mast cell degranulation without involvement of the intracellular receptor. Increased understanding of AMPs mechanism of action, their interaction with non-target cells and low cost production should facilitate further clinical development.

## MATERIALS AND METHODS

All cell culture reagents and pertussis toxin were purchased from Invitrogen (Gaithersburg, MD). Amaxa transfection kit (Kit V) (Lonza, Gaithersburg, MD), recombinant human cytokines (Peprotech, Rocky Hill, NJ), C3a (Complement Technology, Tyler, TX), RC-100 (VWR Bachem, Louisville, KY) and Protegrin 1 (Anaspec, Freemont, CA) were purchased from the sources indicated.

### Culture of mast cell lines

LAD2 human mast cells were maintained in complete StemPro-34 medium supplemented with 100 ng/ml hSCF [89]. Rat basophilic leukemia (RBL-2H3) cells were maintained as monolayer cultures in Dulbecco's modified Eagle's medium (DMEM) supplemented with 10% FBS, L-glutamine (2 mM), penicillin (100 IU/ml) and streptomycin (100 mg/ml) [90].

### Transfection of RBL-2H3

RBL-2H3 cells were transfected with plasmids encoding hemeagglutinin (HA)-tagged MrgX2 using the Amaxa nucleofector device and Amaxa kit V according to the manufacturer's protocol. Following transfection, cells were cultured in the presence of G-418 (1 mg/ml) and cells expressing equivalent receptors were sorted using an anti-HA specific antibody 12CA5/FITC-conjugated anti-mouse-IgG and subsequently used for studies on degranulation and confocal microscopy.

### Calcium mobilization

Ca^2+^ mobilization was determined as described previously [90]. Briefly, LAD2 cells (0.2 × 10^6^) were loaded with indo-1 AM (1 μM) for 30 min at room temperature. Cells were washed and resuspended in 1.5 ml of HEPES-buffered saline. Ca^2+^ mobilization was measured in a Hitachi F-2500 spectrophotometer with an excitation wavelength of 355 nm and an emission wavelength of 410 nm.

### Degranulation

LAD2 cells (5 × 10^3^) and RBL-2H3 cells (5 × 10^4^) were seeded into 96-well plates in a total volume of 50 μl HEPES buffer containing 0.1% BSA and exposed to the indicated peptides. In some assays cells were pretreated with pertussis toxin (PTx, 100 ng/ml, 16 h). For total β-hexosaminidase release, unstimulated cells were lysed in 50 μl of 0.1% Triton X-100. Aliquots (20 μl) of supernatant or cell lysate were incubated with 20 μl of 1 mM p-nitrophenyl-N-acetyl-β-D-glucosamine for 1.5 h at 37°C. Reaction was stopped by adding 250 μl of a 0.1 M Na_2_CO_3_/0.1 M NaHCO_3_ buffer and absorbance was measured at 405 nm [90].

### Biomass production and immunoblot analysis of AMPs from transgenic plants

Seeds from transplastomic plants expressing GFP-RC101 and GFP-PG1 [28] were germinated on Murashige and Skoog medium containing 500 mg/l spectinomycin. After confirmation of GFP fluorescence, transgenic plants expressing AMPs were transferred to pots in the greenhouse for growth under controlled light, temperature and moisture. Because the psbA sequence used for the transgene expression is regulated by light mature leaves were harvested at 6 PM for maximizing protein accumulation. To determine GFP protein expression levels Bradford assay was performed on plant extracts and purified proteins to find the total soluble protein (TSP) concentration. Based on this, appropriate dilutions were made and mixed with 5X sample buffer. The GFP standard and samples were boiled for 3 minutes and loaded onto a 1.0 mm thick 12% SDS-PAGE gel for electrophoresis. The separated proteins were then transferred onto nitrocellulose membrane at 85V for 45 min. The nitrocellulose membrane was then blocked with PTM (1X PBS, 0.05% Tween-20, 3% Milk) for 1 h at room temperature. Mouse anti-GFP primary antibody (EMD Millipore, cat# MAB 3836, 1:3,000) was added in PTM and incubated overnight at 4°C. The membrane was washed 3 times with PBS-T (1X PBS, 0.05% Tween-20), 10 min each time. Goat anti-mouse secondary antibody (Southern Biotech, cat# 1031-05, 1:4,000) was added in PTM and incubated for 2 h at room temperature. Membrane was developed using SuperSignal West Pico chemiluminescent substrate (Thermo Scientific, cat# 34079).

### Purification of AMPs from plants

The GFP-PG1, GFP-RC101 and control GFP peptides were purified as described previously [28]. One gram of finely ground frozen leaf material was suspended in 5 ml of protein extraction buffer (100 mM NaCl, 10 mM EDTA, 200 mM Tris HCl pH 8, 0.2% [v/v] Triton X-100, 400 mM sucrose, and 2 mM phenylmethylsulfonyl fluoride, 1X solution of Pierce protease inhibitor cocktail). Vortexed samples were sonicated and centrifuged at 2500 × g for 5 min. The lysate was then subjected to the organic extraction protocol described by Skosyrev et al [91]. Saturated ammonium sulfate solution was added to the cleared lysate so that the final lysate contained 70% ammonium sulfate [v/v]. The precipitated proteins were then extracted into 100% ethanol by adding 1/4^th^ and 1/16^th^ of the volume of the lysate/ammonium sulfate mixture (aqueous phase) followed by shaking for 2 min and centrifugation at 2500 × g for 5 min. The ethanol extracts were pooled and to 1/3^rd^ volume of this ethanol phase 5M NaCl was added to make a final concentration of 1.6 M. The aqueous phase was collected and salt was removed using a desalting column (Thermo Scientific, Zeba spin column, 7 kDa MWCO). The desalted aqueous phase was loaded onto the Butyl-Toyopearl Hydrophobic Interaction Column (TOSOH Butyl-650S resin, Grade S resin, particle size 65 μm, Column Volume 48 ml) pre-equilibrated with 20% ammonium sulfate in 10 mM Tris-HCl, 10 mM EDTA pH 7.8. Subsequently, the column was washed with 3 column volume (CV) of the equilibration buffer and 2 CV with 10% step wise reduction in ammonium sulfate. Proteins were eluted with salt free elution buffer containing 10 mM Tris-HCl, 10 mM EDTA pH 7.8. The proteins were then dialyzed three times in dialysis buffer containing 1 mM NaCl, 0.1 mM Tris-HCl, and 10 μM EDTA, pH 8.0. Dialyzed proteins were shell frozen in a freezing bath and lyophilized. The purified proteins were then reconstituted in a small volume of reconstitution buffer (PBS, 10% glycerol).

### Fluorescence gel and fluorescence intensity readings

Total soluble protein (TSP) concentration was determined by the Bradford assay, and different quantities of protein were loaded with non-denaturing sample buffer (50 mM Tris-HCl, pH 6.8, 2% SDS, 0.04% Bromophenol blue, 10% glycerol) into a 12% native polyacrylamide gel. After electrophoresis, gels were observed under UV light for GFP fluorescence. Images were taken and analyzed with Image J software. For fluorescence intensity calculations, the Gemini EM microplate reader was used and calculations were done with the Softmax Pro software (Molecular Devices). Standard GFP (Millipore, cat# 14-392) was used to calculate GFP concentrations in samples based on the fluorescence intensity. Samples and GFP standards (standard curve range from 31.25 ng to 500 ng) were dissolved in 200 μl of 10 mM Tris-HCl pH 8.0 and fluorescence intensities were measured by exciting the samples at 400 nm and measuring emission at 510 nm using a 96-well optical bottom plate. For Commassie staining gels were soaked in fixing solution (40% ethanol, 10% acetic acid in Milli Q water) for 20 min at room temperature on a shaker. Subsequently, the gel was stained overnight in colloidal Coomassie G-250 stain (35% methanol, 2.5% phosphoric acid and 10% ammonium sulfate in Milli Q water) and destained first by rinsing with water three times followed 10% acetic acid.

### Antimicrobial assay

Antimicrobial assay was adapted from the microbroth dilution assay described by Steinberg and Lehrer [92]. An overnight culture of E coli grown in tryptic soy broth (TSB) was diluted to 1:20 in the same media and OD_600_ was measured using the Nanodrop 2000C spectrophotometer. From the measured OD value the bacterial concentration in terms of colony forming units (CFU)/ml was calculated based on the formula provided below.

A bacterial density of 4 × 10^5^ CFU/ml was used as the starting inoculum. Bacterial suspension (250 μl) was grown in the presence of AMPs at the indicated final concentrations on a shaker (VWR Benchtop Shaking Incubator, Model 1570) at 240 RPM and 37°C. The growth curves of these cultures were followed for 8 h by measuring OD_600_ at 2 h intervals. At the end of 8 h the cultures were diluted and plated on LB agar to determine CFU the next day.

### Confocal microscopy

Mock and MrgX2-transfected RBL-2H3 cells were grown on coverslips and maintained in fresh complete media. Cells were incubated with control GFP and PG1-GFP (4 μg/ml) for 30 min in 50 μl HEPES buffer containing 0.1% BSA, washed with ice cold PBS and then fixed in 4% paraformaldehyde solution. Confocal microscopy was performed on a Nikon A1R laser scanning confocal microscope with a 60x water objective (NA 1.2), using a fluorescein isothiocyanate filter with the emission wavelength of 488 nm. Images were analyzed using Nikons Elements 4.1 software.
